# Porous Medium Equation in Graphene Oxide Membrane: Nonlinear Dependence of Permeability on Pressure Gradient Explained

**DOI:** 10.3390/membranes11090665

**Published:** 2021-08-29

**Authors:** Lukáš Mrazík, Pavel Kříž

**Affiliations:** 1 Department of Computing and Control Engineering, University of Chemistry and Technology Prague, 166 28 Prague, Czech Republic; 2 Department of Mathematics, University of Chemistry and Technology Prague, 166 28 Prague, Czech Republic; krizp@vscht.cz

**Keywords:** nonlinear diffusion, graphene oxide, porous medium equation, permeability, selectivity, membrane gas separation

## Abstract

Membrane performance in gas separation is quantified by its selectivity, determined as a ratio of measured gas permeabilities of given gases at fixed pressure difference. In this manuscript a nonlinear dependence of gas permeability on pressure difference observed in the measurements of gas permeability of graphene oxide membrane on a manometric integral permeameter is reported. We show that after reasoned assumptions and simplifications in the mathematical description of the experiment, only static properties of any proposed governing equation can be studied, in order to analyze the permeation rate for different pressure differences. Porous Medium Equation is proposed as a suitable governing equation for the gas permeation, as it manages to predict a nonlinear behavior which is consistent with the measured data. A coefficient responsible for the nonlinearity, the polytropic exponent, is determined to be gas-specific—implications on selectivity are discussed, alongside possible hints to a deeper physical interpretation of its actual value.

## 1. Introduction

Membrane gas separation is a technology with a well established market [1] and understanding the flow of gases through membranes is therefore of crucial importance. Researchers are competing in surpassing the Robeson upper bound [2] by developing new materials and fabrication processes [3,4,5,6,7,8,9,10]; the other direction of research aims at a deeper understanding of mechanisms of transport of gases through the membranes.

To properly classify a membrane, two main factors are considered: permeance (gas permeation rate per membrane area per pressure difference) or permeability (permeance per membrane thickness) and selectivity (ratio of permeances or permeabilities for a pair of gases) [11,12]. This approach implicitly assumes at least one of the following: (i) the system is linear in normalized parameters, namely membrane area, membrane thickness, and pressure difference; (ii) any nonlinearities in said parameters will cancel out; (iii) the nonlinearity is negligible in the operating range of the system. This is a perfectly valid approach as long as a false implication is not assumed, that by determining selectivity one is free to consider the measured permeability as a linear function of the pressure difference.

Graphene oxide (GO) membranes are studied extensively for their unique mechanical properties—ultimate thinness, flexibility, chemical stability, and mechanical strength [3,13], as well as great separation properties—they offer enormous gas permeation rates while maintaining decent selectivity [14,15,16,17,18] not to mention many other applications beyond gas separation [19,20,21,22,23,24,25].

Studying the properties of the derivatives of GO membranes has been the leading trend recent years. GO is mixed with other substances such as polyether block amide polymer (PEBAX) [26], bio-based polyurethane [27], hydrophilic poly(2-acrylamido-2-methyl propane sulfonic acid) (PAMS) [28], MIL-101(Cr) nanocrystals [29], gold nanoparticles [30], or carbon nanotubes [31]. GO membranes for gas separation applications have been thoroughly reviewed in 2019 [32].

Various approaches of modeling the transport mechanisms inside graphene/GO have been reported. The most common are molecular dynamic simulations with an implementation of pores geometry and energy barriers [33,34,35,36], accompanied by adsorption and direct penetration in terms of statistical thermodynamics [37] and molecular sieving [38]. The pressure dependence is seldom addressed and if so, then only as a linear parameter of the simulation intended to be normalized.

Our measurements of gas permeability of a GO membrane [3] within a manometric integral permeation apparatus [39] show nonlinear dependence of gas permeability on input pressure, invalidating any linear partial differential equation (PDE), or any other modeling technique regarding the pressure as normalizable, as a proper governing equation/model for the gas transport through the membrane.

In this manuscript we aim to (i) report the observation of the above mentioned nonlinearity; (ii) provide a criterion for a PDE/model of gas transport through a GO membrane to be able to describe the nonlinearity; (iii) propose a *Porous Medium Equation* (PME) as a candidate for a governing equation. PME is rather a class of nonlinear PDEs of a given form—we derive two specific PMEs for pressure and resp. density in Appendix A to provide mathematical context.

We describe the dynamics of the experiment from the point of view of the integral cell of the apparatus and based on law of conservation of mass we reformulate the dynamics in terms of pressure and density inside the membrane. We introduce some reasoned assumptions and simplifications in order to make it possible to study the pressure and density profiles within the membrane in their stationary state, without actually considering any specific PDEs as governing equations.

The profiles are solutions to a boundary value problem (BVP) corresponding to the specific experimental setting. We simulate the observation of nonlinearity by introducing a multiplicative change in input pressure and calculating the impact on the gas permeation rate—only at this moment the two PMEs and their 1-D stationary solutions of said BVPs are introduced. For the sake of completeness we present analytical solutions to BVPs on a finite domain for Dirichlet and Neumann boundary conditions and means of rewriting mixed boundary conditions as either of them.

Evaluating the consequences of the multiplicative change in input pressure results in a convincingly accurate power-law formula capable of describing the experimental data. This is the key argument in favor of considering PME as a valid governing equation for gas transport through GO membranes. We discuss the implications of the nonlinearity on selectivity, as well as a possible interpretation of the polytropic exponent—the coefficient responsible for the nonlinearity of PMEs.

## 2. Materials and Methods

### 2.1. Manometric Integral Permeameter

**Figure 1:**
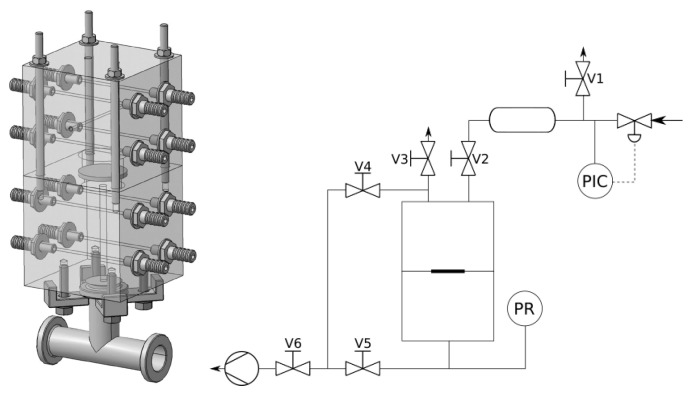
Manometric integral permeameter—(left) AutoCAD model of the cells and tubing, (right) technological scheme of the whole apparatus.

The main component of the apparatus is a stainless-steel construction of two cells with a membrane in between them, for the 3-D model and technological scheme see Figure 1. The top one (source cell) supplies the gas—it is connected to a buffer tank with a pressure controller. The bottom one (integral cell) is equipped with a capacitive pressure gauge. The membrane is placed on a porous sintered steel plug which acts as a porous support for the membrane. Both halves can be connected via a system of valves to a rotary oil pump so that it is possible to evacuate the device. Both cells are water-tempered at 25 °C via total of eight horizontal tubes. The experiment is controlled by six valves labeled V1–V6.

To make a single gas permeability measurement, both cells are evacuated, then a specific gas at specific (controlled) input pressure is supplied into the source cell. As the gas flows through the membrane an increase in pressure inside the integral cell is detected. After a given criteria is met (time elapsed or gauge maximum limit reached) the experiment is terminated.

### 2.2. Membrane

The preparation method for GO is reported in [3], the specific membrane manufacturing process was described as follows.

A volume corresponding to 82 mg of GO was taken from the stock suspension with a GO concentration of 20 mg/mL. The GO suspension was diluted with distilled water to a volume of 25 mL and sonicated in an ultrasonic tray (37 kHz, 300 W) for 15 min. The diluted GO suspension was placed in a pressurized filter cell equipped with a polyamide filter membrane. Filtration was performed using compressed air at a pressure of 3–5 bar. After filtration, the still wet GO membrane was moved (still with the polyamide support) to a desiccator with a constant 30% relative humidity (provided by saturated ${\mathrm{MgCl}}_{2}$ solution at 20 °C). In this environment the membrane was stored for at least 24 h in order to balance the moisture in the membrane.

## 3. Mathematical Formulation of the Experiment

The mathematical goal is to develop a criterion any PDE must fulfill in order for it to be able to describe the observed nonlinearity. The derivation is done in general setting—PME is not considered as a specific mathematical model for our experiment in this section.

The complete mathematical description of the experiment leads to a (non)linear evolution equation with appropriately chosen initial and boundary conditions. Unfortunately, such problem can hardly be solved analytically. Hence, a few simplifications are adopted (described below including the reasoning) so that analytical solutions are available. The main idea is to split the solution into two parts: (i) solution in time domain in the integral cell (assumed to be linear at the beginning of the experiment) and (ii) solution in space domain in the membrane (assumed to be stationary very soon). The two solutions are then linked via law of conservation of mass.

### 3.1. Linearity of Single Measurement in the Integral Cell

In this section we claim that in the range of detected pressures the measured response can be approximated as a linear function of time.

Measurements as described in Section 2.1 are a standard experimental identification technique of measuring step responses. The available range of input pressure $p_{in}$ is given by the safety limits of gas tubing and ranges from 1 to 4 bar; the range of our pressure gauge is 10 Torr = 1333 Pa. This means that it is possible to measure approximately only the first L=0.0133, resp. L=0.003325 of the response. In this range the response is almost a linear function of time, so the only extractable information is its slope—the pressure time difference as a ratio $\frac{p_{end}}{t_{end}}$.

The pressure difference changes slightly during the measurement—to vindicate the negligibility of the effect it can be roughly bounded by comparing a linear function with a standard unit step response of first order system,

(1) $$y_{\mathrm{lin}}=t\;∼\;y_{\mathrm{step}}=1-e^{-t},\;t\in \left(0,\varepsilon \right),$$

where the first order system is the only possible long-term-behavior-explaining approximation possible, provided we only the slope is measured. Let the unit step response hit the limit value *L*,

(2) $$y_{\mathrm{step}}\left(t_{L}\right)=L\Leftrightarrow t_{L}=-\log\left(1-L\right).$$

At that time the differences in values and derivatives are

(3) $$\Delta y=y_{\mathrm{step}}\left(t_{L}\right)-y_{\mathrm{lin}}\left(t_{L}\right)=-L-\log\left(1-L\right),\;\Delta y^{\prime }=y_{\mathrm{step}}^{\prime }\left(T\right)-y_{\mathrm{lin}}^{\prime }\left(T\right)=L,$$

which for the greatest achievable limit value L=0.0133 yields $\Delta y\approx 9\times 10^{-5}$ and for the lowest achievable limit value L=0.0026 yields $\Delta y\approx 5.6\times 10^{-6}$. Furthermore, the reported data never actually got even close to the gauge limit—the maximum value of *L* is another order of magnitude lower at L=0.000126, which corresponds to the upper bound of the error being less then than 1‰, which is indeed negligible in comparison with other effects such as the input pressure deviating from the Heaviside function, the precision of membrane thickness measurement, or even the homogeneity of the thickness.

### 3.2. Nearly-Stationary Behavior within the Membrane

In this section we claim that if the measured response is a linear function of time, then the pressure profile inside membrane is close to stationary state.

The measured response of the system should be slightly delayed, as it takes some time for the gas to actually move all the way through the membrane and to the gauge. An initial delay can be observed; however, it cannot be distinguished between a delay caused by membrane, by the gauge distance, and by control timing uncertainties. Rather a few seconds are excluded from the evaluation, until the profile becomes linear.

The fact that the time profile becomes linear and stays linear for the measured time interval hints strongly towards an idea that the inner dynamics of pressure distribution inside the membrane is much faster than the dynamics of filling of the integral cell.

We claim that for sufficiently large time t>ε the shape of the profile in the membrane is nearly static, otherwise a different than linear time dependence would occur; an argument supported also by comparing sheer volumes—the membrane is three orders of magnitude smaller in volume than the integral cell.

### 3.3. Conservation of Mass on the Membrane/Integral Cell Interface

In this section the goal is to express the measured quantity, pressure time derivative inside the integral cell—we do that by comparing the mass accumulation inside the integral cell from the point of view of ideal gas law and the gas flux through membrane.

From the ideal gas law the mass inside the integral cell can be expressed as

(4) $$m_{IC}=\frac{\bar{p_{IC}}V_{IC}M}{RT},$$

where the subscript ${□}_{IC}$ stands for property of the integral cell and $\bar{p_{IC}}$ is the mean pressure. Assuming that only mass and pressure can change, taking the time derivative yields 


(5) $$\frac{\partial m_{IC}}{\partial t}=\frac{V_{IC}M}{RT}\frac{\partial \bar{p_{IC}}}{\partial t}.$$

There is only one way of changing the mass inside the integral cell—by gas flowing through the membrane. This provides another way of expressing the mass accumulation, commonly described in terms of surface integral of mass flow through the boundary,

(6) $$\frac{\partial m_{IC}}{\partial t}={∯}_{\partial V_{IC}}\rho \vec{q}\cdot \vec{n}\;dS.$$

The cell is assumed to be perfectly insulated, so the only part of the boundary with nonzero flux is the membrane with surface $A_{M}$. If we think of the membrane as being perpendicular to the *x*-axis and localized at x∈(0,w), then the integral cell can be localized at $x\in \left(w,w+\frac{V_{IC}}{A_{M}}\right)$ by introducing a cylindrical simplification of the cell. From the simplification it follows that there is no radial flux anywhere, allowing us to simplify and evaluate the integral,

(7) $$∯{}_{\partial V_{IC}}\rho \vec{q}\cdot \vec{n}\;dS=A_{M}\lim_{x\to w_{+}}\left(\rho q_{x}\right).$$

The limit stresses our point of view of the integral cell still being considered. Based on conservation of mass, we can switch the point of view and state that whatever comes inside the integral cell must have come from the membrane, where all the necessary tools are at hand. Introducing Darcy’s law of diffusion,

(8) $$q_{x}=-\frac{k}{\mu }\lim_{x\to w_{-}}\frac{\partial p}{\partial x}$$

where the left-sidedness of the limit switches the point of view to that of the membrane. Equation (7) can be than rewritten in terms of the properties of the membrane,

(9) $$A_{M}\lim_{x\to w_{+}}\left(\rho q_{x}\right)=-A_{M}\frac{k}{\mu }\lim_{x\to w_{-}}\left(\rho \frac{\partial p}{\partial x}\right).$$

Combining the two expressions of time change of mass, first derived from ideal gas law (5), second from the surface integral (9) the pressure time derivative can be expressed,

(10) $$\begin{array}{cc} \; & \frac{\partial m_{IC}}{\partial t}=V_{IC}\frac{M}{RT}\frac{\partial \bar{p_{IC}}}{\partial t}=-\frac{k}{\mu }A_{M}\lim_{x\to w_{-}}\left(\rho \frac{\partial p}{\partial x}\right),\\ & \frac{\partial \bar{p_{IC}}}{\partial t}=-\frac{k}{\mu }\frac{RT}{M}\frac{A_{M}}{V_{IC}}\lim_{x\to w_{-}}\left(\rho \frac{\partial p}{\partial x}\right). \end{array}$$

The key observation here is that the measured time-dependent increase of pressure is proportional to the density and pressure gradient at the bottom of the membrane.

### 3.4. Boundary Conditions for Pressure Profile in the Membrane

In this section the goal is to interpret the experiment as a boundary value problem, whose stationary solution will be further investigated.

The focus is now on the membrane itself. The boundary condition at x=0 is fairly simple to implement—the pressure is set and controlled there, thus a pressure Dirichlet boundary condition is prescribed.

As it was already hinted by the near-stationary state argument, we will not be predicting the whole dynamics. We rather prescribe the second boundary condition as static and focus on the consequences of changing the input pressure.

To get the stationary solution an actual condition must be of course prescribed at x=w. To be as close as possible to the actual measurement process, we prescribe a general Dirichlet condition $p_{out}$ with the intention of taking the limit $p_{out}\to 0_{+}$, if required.

### 3.5. Representation of the Nonlinearity

The final goal is to formalize a method of PDE validation via the stationary solutions’ capability of predicting the observed nonlinearity of the relation between input pressure and gas permeation rate.

We will work around the dynamics by exploiting Equation (10)—if two different situations can be described, one for input pressure $p_{in}$ and second for a different input pressure $\xi p_{in}$, by comparing their values of $\rho (w_{-})$ and $\frac{\partial p}{\partial x}\left(w_{-}\right)$ the dependence of $\frac{\partial p_{IC}}{\partial t}$ on the multiplicative factor ξ can be predicted.

Let *F* be a function of $p_{in}$ returning the value predicted by (10) for a general $p_{out}$,

(11) $$F\left(p_{in}\right)=-\frac{k}{\mu }\frac{RT}{M}\frac{A_{M}}{V_{IC}}\lim_{x\to w_{-}}\rho \frac{\partial p}{\partial x},$$

where the ρ and $\frac{\partial p}{\partial x}$ are derived from the solution to the BVP $p\left(0\right)=p_{in},p\left(w\right)=p_{out}$. There are two possibilities of how to introduce the multiplicative factor—either as a BVP $p\left(0\right)=\xi p_{in}$, $p\left(w\right)=p_{out}$ or as a BVP $p\left(0\right)=\xi p_{in},p\left(w\right)=\xi p_{out}$. The former case would be a better representation of an experiment terminated by reaching the gauge limit, the latter case would correspond better to a termination-by-time-elapsed experimental setting. We will solve the latter and at the end show a solution to the former. Comparing the solutions with and without ξ-factor should predict (hopefully nonlinear) dependence,

(12) $$\frac{F(\xi p_{in})}{F(p_{in})}=f\left(\xi \right),$$

which for linear case would simply read f(ξ)=ξ.

### 3.6. Numerical Simulation Support for the Assumptions

To further support the claims and assumptions, most importantly the nearly-stationary behavior within the membrane, we performed numerical simulations of the membrane and integral cell via PME and found them consistent with both the presence and the shape of the nearly-stationary state within the membrane, as well as with the observed nonlinear dependence of gas permeation rate on input pressure [40].

Using Finite Volume Method the complete dynamics of the system consisting of the membrane and the integral cell was simulated. The geometry was slightly more complicated, as the integral cell was modeled as a coaxial cylinder but with twice the radius of the membrane—the rotational symmetry has been conserved. The single slice being rotated had the inner angle of 2°, five control volumes alongside the membrane radius, and resp. ten control volumes alongside the integral cell radius. Along the common axis the membrane was divided into 50 slides of constant width, the integral cell had 100 slides, linearly increasing their width to fit the actual volume of the integral cell.

The governing equation inside the membrane was derived from the divergence form of general PME, $\frac{\partial u}{\partial t}=D\Delta \cdot \left(\Gamma {\left|u\right|}^{\Gamma -1}\nabla u\right)$, in the integral cell a linear form of the equation (Γ=1) with diffusion coefficient of nine orders of magnitude higher has been at place.

For the temporal domain, equidistant Crank–Nicolson implicit scheme with the forward weight of 0.9 and time step Δt=0.1s was utilized. The implicit part of the scheme took only the gradient (∇u) into consideration, as calculating the nonlinear part $\left({\left|u\right|}^{\Gamma -1}\right)$ implicitly would be a disproportionately complex problem to solve.

## 4. Stationary Solutions of Porous Medium Equation

Properly derived in the Appendix A, we present three formulations of PME for each quantity in Table 1.

PME has been thoroughly studied by Vazquez [41]. In Chapter 4.1—*Some very simple solutions* he describes 1-D stationary solutions of PME. The actual derivation is rather tedious, highly repetitive, and not of any particular interest, so they are presented in a factual manner.

The mixed BVPs have no explicit solution for general γ, as it contains a root of a fractional polynomial, thus only the system of equations to be solved is presented in Table 2, to be used to rewrite the mixed problem as either Dirichlet or Neumann. Solutions to Dirichlet BVPs are presented in Table 3 and to the Neumann BVPs in Table 4: each solution consists of two profiles, p(x) and ρ(x).

For an in-depth mathematical discussion see [41], particularly Chapter 20.1—*Large-time behavior of the GDP, Non-negative solutions* for the convergence analysis, and corresponding parts of Chapters 5–8 for the uniqueness analysis.

### Nonlinearity Predicted

Once the analytical solutions of stationary BVPs of PME are known, it is possible to calculate the (nonlinear) prediction of the gas permeation rate dependence on input pressure, as described in Section 3.5.

Take the density solution and the derivative of pressure solution to Dirichlet BVP $p_{in}$ and $p_{out}$,

(13) $$\begin{array}{cc} \rho & ={\left(\frac{p_{in}}{e_{0}}\right)}^{\frac{1}{\gamma }}{\left(1-\frac{x}{w}\left(1-{\left(\frac{p_{out}}{p_{in}}\right)}^{\frac{\gamma +1}{\gamma }}\right)\right)}^{\frac{1}{\gamma +1}},\\ \frac{\partial p}{\partial x} & =-\frac{p_{in}}{w}\frac{\gamma }{\gamma +1}\left(1-{\left(\frac{p_{out}}{p_{in}}\right)}^{\frac{\gamma +1}{\gamma }}\right){\left(1-\frac{x}{w}\left(1-{\left(\frac{p_{out}}{p_{in}}\right)}^{\frac{\gamma +1}{\gamma }}\right)\right)}^{-\frac{1}{\gamma +1}}. \end{array}$$

When multiplied together, the whole term with spatial coordinate actually cancels out, making the limit calculation rather trivial,

(14) $$\lim_{x\to w_{-}}\rho \frac{\partial p}{\partial x}=-{\left(\frac{p_{in}}{e_{0}}\right)}^{\frac{1}{\gamma }}\frac{p_{in}}{w}\frac{\gamma }{\gamma +1}\left(1-{\left(\frac{p_{out}}{p_{in}}\right)}^{\frac{\gamma +1}{\gamma }}\right)=-\frac{\gamma }{\gamma +1}\frac{1}{e_{0}w}\left(p_{in}^{\frac{\gamma +1}{\gamma }}-p_{out}^{\frac{\gamma +1}{\gamma }}\right).$$

Plugging the evaluated limit into (11), the prediction of the gas permeation rate yields

(15) $$F\left(p_{in}\right)=-\frac{k}{\mu }\frac{RT}{M}\frac{A_{M}}{V_{IC}}\lim_{x\to w_{-}}\rho \frac{\partial p}{\partial x}=\frac{k}{\mu }\frac{RT}{M}\frac{A_{M}}{V_{IC}}\frac{\gamma }{\gamma +1}\frac{1}{e_{0}w}\left(p_{in}^{\frac{\gamma +1}{\gamma }}-p_{out}^{\frac{\gamma +1}{\gamma }}\right).$$

Now compare the prediction after introducing multiplicative factor ξ as in (12),

(16) $$f\left(\xi \right)=\frac{F(\xi p_{in})}{F(p_{in})}=\frac{\frac{k}{\mu }\frac{RT}{M}\frac{A_{M}}{V_{IC}}\frac{\gamma }{\gamma +1}\frac{1}{e_{0}w}\left({\left(\xi p_{in}\right)}^{\frac{\gamma +1}{\gamma }}-{\left(\xi p_{out}\right)}^{\frac{\gamma +1}{\gamma }}\right)}{\frac{k}{\mu }\frac{RT}{M}\frac{A_{M}}{V_{IC}}\frac{\gamma }{\gamma +1}\frac{1}{e_{0}w}\left(p_{in}^{\frac{\gamma +1}{\gamma }}-p_{out}^{\frac{\gamma +1}{\gamma }}\right)}=\xi^{\frac{\gamma +1}{\gamma }}.$$

Resorting back to the ambiguity of whether or not to multiply the output pressure by ξ as well, in that case we get

(17) $$\frac{\hat{F}\left(\xi p_{in}\right)}{\hat{F}\left(p_{in}\right)}=\frac{\frac{k}{\mu }\frac{RT}{M}\frac{A_{M}}{V_{IC}}\frac{\gamma }{\gamma +1}\frac{1}{e_{0}w}\left({\left(\xi p_{in}\right)}^{\frac{\gamma +1}{\gamma }}-p_{out}^{\frac{\gamma +1}{\gamma }}\right)}{\frac{k}{\mu }\frac{RT}{M}\frac{A_{M}}{V_{IC}}\frac{\gamma }{\gamma +1}\frac{1}{e_{0}w}\left(p_{in}^{\frac{\gamma +1}{\gamma }}-p_{out}^{\frac{\gamma +1}{\gamma }}\right)}=\frac{{\left(\xi p_{in}\right)}^{\frac{\gamma +1}{\gamma }}-p_{out}^{\frac{\gamma +1}{\gamma }}}{p_{in}^{\frac{\gamma +1}{\gamma }}-p_{out}^{\frac{\gamma +1}{\gamma }}},$$

which is much harder to cope with. However, as $p_{out}$ is negligible to $p_{in}$, taking the limit $p_{out}\to 0_{+}$,

(18) $$\hat{f}\left(\xi \right)=\lim_{p_{out}\to 0_{+}}\frac{{\left(\xi p_{in}\right)}^{\frac{\gamma +1}{\gamma }}-p_{out}^{\frac{\gamma +1}{\gamma }}}{p_{in}^{\frac{\gamma +1}{\gamma }}-p_{out}^{\frac{\gamma +1}{\gamma }}}=\xi^{\frac{\gamma +1}{\gamma }},$$

results in exactly the same expression.

## 5. Results

### 5.1. General Setting

**Figure 2:**
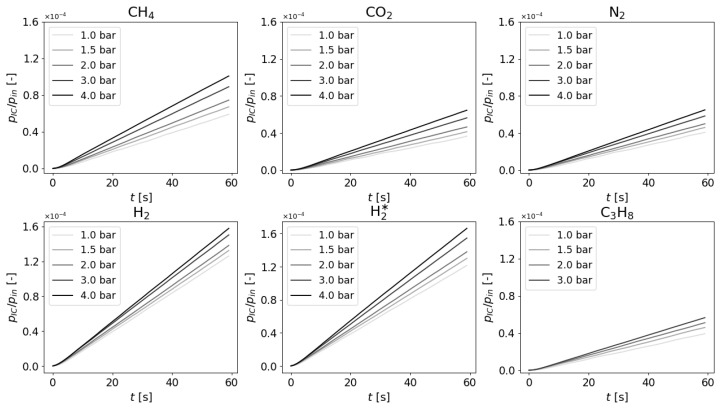
Measurements of increasing pressure detected in the integral cell of manometric integral permeameter, normalized by input pressure. Without the nonlinearity, measurements would be expected to align.

**Figure 3:**
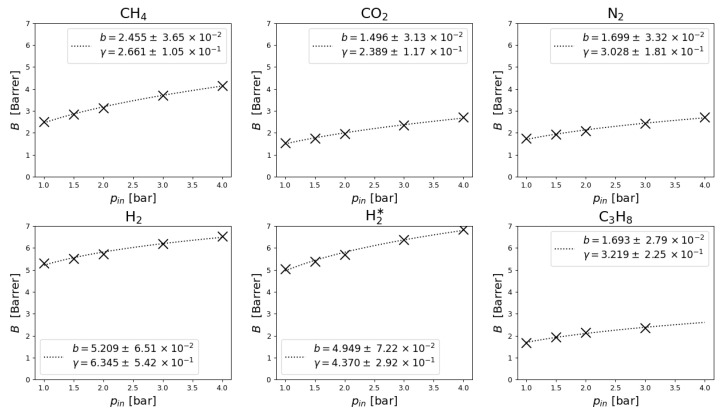
Measurements of gas permeability $\left(B\right)$ dependence on input pressure $\left(p_{in}\right)$ and nonlinear power-law regression of measured data. Without the nonlinearity, measurements would be expected to be constant (not only straight line, as the data are normalized).

The volume of integral cell of the apparatus was determined via a combination of geometric calculations and datasheet values as $V_{IC}=42.81\pm 0.005\;{\mathrm{cm}}^{3}$. All presented data was measured on a single membrane which without the polyamide support had a thickness of w=29.5±0.7μm and effective area $A_{M}$ of a circle with diameter of 3 cm. Its gas permeability was low enough in terms of pressure gauge limit, thus the criterion for experiment termination was passing of $t_{end}=60\;s$. In Figure 2 we present normalized measurements of $p_{IC}$ in time and in Figure 3 we present gas permeability [42] dependence on input pressure $B\left(p_{in}\right)$, calculated by

(19) $$B=\frac{\partial p_{IC}}{\partial t}\frac{V_{IC}}{RT}\frac{w}{A_{M}p_{in}},\;\left[\mathrm{Barrer}\right]=3.35\times 10^{-16}\left[\frac{\mathrm{mol}}{s\cdot m\cdot \mathrm{Pa}}\right].$$

Every line, resp. every point is an average of usually four distinct realizations of the experiment.

### 5.2. Measurements and Data Regression

In Figure 2, a slight delay of the system reaction can be observed, due to which the determination of slope has been postponed by 10 s. In case of linear dependence of gas permeability on input pressure all the curves would coincide (due to normalization by $p_{in}$); however, there is a clear upward trend. The 4 bar propane measurement is excluded as the membrane started to clog itself. There are two measurements of H${}_{2}$, the second is denoted as H${}_{2}^{*}$ and the latter measurement was performed after the propane clogging. The clogging seems to have changed the internal structure of the membrane in such a way that repeatability has been compromised, thus the measurements of C${}_{3}$H${}_{8}$ and H${}_{2}^{*}$ should not be compared with the other four.

In Figure 3 the nonlinearity of gas permeability dependence on input pressure can be observed directly: after normalization, in the linear case a constant value would be expected, but again an upward trend is present—change in input pressure results in disproportionally greater change of permeability. The dotted line is a nonlinear least square regression of the equation

(20) $$B_{\mathrm{gas}}\left(b,\gamma \right)=b\xi^{\frac{1}{\gamma }},$$

which is the power-law representation of the nonlinearity predicted by PME, as a set-point the gas permeability at $p_{in}=1\;\mathrm{bar}$ was used. It results in a pair of parameters—*b* being the predicted gas permeability at $p_{in}$ and γ being the polytropic exponent.

### 5.3. Polytropic Exponent Interpretation

For each gas, a pair of parameters $\left(b,\gamma \right)$ is obtained. The polytropic exponent can be further interpreted as a molar heat capacity,

(21) $$\gamma =\frac{c-c_{p}}{c-c_{V}}\Rightarrow c=c_{p}-\frac{\gamma R}{\gamma -1}=c_{V}-\frac{R}{\gamma -1},$$

resulting in values in Table 5.

## 6. Discussion

### 6.1. Porous Medium Equation Capability of Describing Observed Nonlinearity

We have presented nonlinear gas permeability measurements in dependence on input pressure. Under some assumptions, most notably the nearly-stationary behavior within the membrane, the observed nonlinearity was described, as can be most clearly seen in Figure 3—any linear PDE would result in a simple constant. In other words, PME turned out to be much more suitable model for gas flow through GO membrane than any linear PDE (such as diffusion equation, for example).

Considering PME as a valid model for gas permeability of GO membranes provides an insight into the inner structure of the membrane, as the membranes should be “porous enough” to be thought of as a porous medium as described by Whitaker in [44].

### 6.2. Nonlinearity Implications for Selectivity

The most significant consequence of the nonlinearity is that for two gases with different polytropic exponent, selectivity as a ratio of gas permeabilities depends on the input pressure used for determining the permeabilities. For selectivity between gases *X* and *Y*, and for set-point permeabilities $b_{X},b_{Y}$ measured at $p_{in}$, the selectivity dependence can be written as

(22) $$\alpha_{XY}\left(\xi \right)=\frac{B_{X}}{B_{Y}}=\frac{b_{X}\xi^{\frac{1}{\gamma_{X}}}}{b_{Y}\xi^{\frac{1}{\gamma_{Y}}}}=\frac{b_{X}}{b_{Y}}\xi^{\frac{1}{\gamma_{X}}-\frac{1}{\gamma_{Y}}},$$

which predicts the selectivity at $\xi p_{in}$. High selectivity is desired, which corresponds to $b_{X}\gg b_{Y}$ but now the nonlinearity also makes it desirable for $\gamma_{X}\ll \gamma_{Y}$ (for ξ>1).

In our data, selectivity tends to decrease with increasing pressure—most noticeably, selectivity based on measurements of H${}_{2}$ and CH${}_{4}$ gives $\alpha_{H_{2},{\mathrm{CH}}_{4}}\left(1\;\mathrm{bar}\right)=3.423$; however, with increased pressure a significant drop occurs with $\alpha_{H_{2},{\mathrm{CH}}_{4}}\left(4\;\mathrm{bar}\right)=2.417$.

By no means are we claiming that our membrane can compete in absolute numbers with state of the art membranes, we mainly want to draw attention to the possible significance of pressure gradient dependence. In industrial applications our results might give insight into the problem of finding an optimal operating point and for researchers the main implication is that points on Robeson plot might not be as static as they seem.

### 6.3. Physical Interpretation of Polytropic Exponent

The molar heat capacities as presented in Table 5 are a direct result of introducing a change of temperature into a system described by the first law of thermodynamics and ideal gas law. Specific interpretation of the molar heat capacities might be possible, if one would describe the membrane from the thermodynamical point of view. Such interpretation could hint to phenomena happening inside the membrane such as sorption; however, further research and measurements are needed.

### 6.4. Pressure Range of the Measurement

Our manometric integral permeameter has an operating range from 1 to 4 bars, possibly to 5 bars if we shrink our safety margins. In this range, only propane measurements exhibited different behavior, clogging the membrane at 4 bars. Other gases might exhibit the same clogging effect at higher pressures—specifying clogging pressures might provide another point of view, e.g., if a correlation with molecular diameter shows up. However, such study would require more experiments and in-depth analysis, which is not in the scope of this paper.

### 6.5. Future Research Outline

We are currently working on reproducing the measured data with a higher resolution of input pressure. Preliminary data show that for a slightly different membrane preparation technique the membranes are at least a bit compressible, which reduces the width but consequently the porosity. To measure the phenomenon we will design a new manometric integral permeameter with a capability of measuring the width of the membrane under pressure to quantify the compressibility and with a higher level of automation.

Introducing compressibility into the equations is the next mathematical step, one which will hopefully result in more insight into the internal structure of the membranes.

## 7. Conclusions

Nonlinear dependence of gas permeability on pressure difference of graphene oxide membranes has been reported. A criterion of any model’s/equation’s capability of describing the nonlinearity has been formulated in terms of a general multiplicative change of input pressure.

Porous Medium Equation has been proposed as a suitable governing equation for the gas flow through a GO membrane based on the analysis of its 1-D stationary solutions on a finite domain and subsequent prediction of the gas permeation rate. The prediction was consistent with the gas permeation rate observed when measuring on the manometric integral permeameter.

Validity of Porous Medium Equation in the measured range provides strong reason to believe that Graphene Oxide membranes are porous in such a way that is required by the definition of the term Porous Medium by [44].

Implications of the nonlinearity were discussed, most notably that selectivity can depend on pressure gradient under which each gas’ permeability was measured, if the two gases in concern exhibit different values of polytropic exponent.

## Figures and Tables

**Table 1:** Overview of PME formulations. Differential operators act on spatial dimensions, where ∇u is the gradient, $\nabla \cdot \vec{u}$ is the divergence, and Δu is the Laplacian.

Form	Pressure	Density
Laplacian	$\frac{\partial p}{\partial t}=\frac{k}{\varepsilon \mu }\;\frac{\gamma^{2}}{\gamma +1}\;p^{\frac{\gamma -1}{\gamma }}\;\Delta \left(p^{\frac{\gamma +1}{\gamma }}\right)$	$\frac{\partial \rho }{\partial t}=\frac{ke_{0}}{\varepsilon \mu }\frac{\gamma }{\gamma +1}\Delta \left(\rho^{\gamma +1}\right)$
Divergence	$\frac{\partial p}{\partial t}=\frac{k}{\varepsilon \mu }\;\gamma \;p^{\frac{\gamma -1}{\gamma }}\nabla \cdot \left(p^{\frac{1}{\gamma }}\nabla p\right)$	$\frac{\partial \rho }{\partial t}=\frac{ke_{0}}{\varepsilon \mu }\gamma \nabla \cdot \left(\rho^{\gamma }\nabla \rho \right)$
Linearized	$\frac{\partial p}{\partial t}=\frac{k}{\varepsilon \mu }\;\left({∥\nabla p∥}^{2}+\gamma p\Delta p\right)$	$\frac{\partial \rho }{\partial t}=\frac{ke_{0}}{\varepsilon \mu }\rho^{\gamma -1}\left(\gamma^{2}{∥\nabla \rho ∥}^{2}+\gamma \rho \Delta \rho \right)$

**Table 2:** Overview of equations used to reformulate mixed BVPs as either Dirichlet or Neumann BVP.

x=0	x=w	Neumann Part	Dirichlet Part
$p_{in}$	$p_{out}^{\prime }$	$p_{in}^{\prime }=p_{out}^{\prime }{\left(\frac{p_{out}}{p_{in}}\right)}^{\frac{1}{\gamma }}$	$p_{out}^{\frac{1}{\gamma }}\left(p_{out}-\frac{\gamma +1}{\gamma }wp_{out}^{\prime }\right)=p_{in}^{\frac{\gamma +1}{\gamma }}$
$p_{in}^{\prime }$	$p_{out}$	$p_{out}^{\prime }=p_{in}^{\prime }{\left(\frac{p_{in}}{p_{out}}\right)}^{\frac{1}{\gamma }}$	$p_{in}^{\frac{1}{\gamma }}\left(p_{in}+\frac{\gamma +1}{\gamma }wp_{in}^{\prime }\right)=p_{out}^{\frac{\gamma +1}{\gamma }}$
$\rho_{in}$	$\rho_{out}^{\prime }$	$\rho_{in}^{\prime }=\rho_{out}^{\prime }{\left(\frac{\rho_{out}}{\rho_{in}}\right)}^{\gamma }$	$\rho_{out}^{\gamma }\left(\rho_{out}-\left(\gamma +1\right)w\rho_{out}^{\prime }\right)=\rho_{in}^{\gamma +1}$
$\rho_{in}^{\prime }$	$\rho_{out}$	$\rho_{out}^{\prime }=\rho_{in}^{\prime }{\left(\frac{\rho_{in}}{\rho_{out}}\right)}^{\gamma }$	$\rho_{in}^{\gamma }\left(\rho_{in}+\left(\gamma +1\right)w\rho_{in}^{\prime }\right)=\rho_{out}^{\gamma +1}$

**Table 3:** Overview of stationary solutions to Dirichlet BVPs.

*u*	$u_{\mathrm{in}}$	$u_{\mathrm{out}}$	u(x)
*p*	$p_{in}$	0	$p_{in}{\left(1-\frac{x}{w}\right)}^{\frac{\gamma }{\gamma +1}}$
ρ	${\left(\frac{p_{in}}{e_{0}}\right)}^{\frac{1}{\gamma }}$	0	${\left(\frac{p_{in}}{e_{0}}\right)}^{\frac{1}{\gamma }}{\left(1-\frac{x}{w}\right)}^{\frac{1}{\gamma +1}}$
*p*	$e_{0}\rho_{in}^{\gamma }$	0	$e_{0}\rho_{in}^{\gamma }{\left(1-\frac{x}{w}\right)}^{\frac{\gamma }{\gamma +1}}$
ρ	$\rho_{in}$	0	$\rho_{in}{\left(1-\frac{x}{w}\right)}^{\frac{1}{\gamma +1}}$
*p*	$p_{in}$	$p_{out}$	$p_{in}{\left(1-\frac{x}{w}\left(1-{\left(\frac{p_{out}}{p_{in}}\right)}^{\frac{\gamma +1}{\gamma }}\right)\right)}^{\frac{\gamma }{\gamma +1}}$
ρ	${\left(\frac{p_{in}}{e_{0}}\right)}^{\frac{1}{\gamma }}$	${\left(\frac{p_{out}}{e_{0}}\right)}^{\frac{1}{\gamma }}$	${\left(\frac{p_{in}}{e_{0}}\right)}^{\frac{1}{\gamma }}{\left(1-\frac{x}{w}\left(1-{\left(\frac{p_{out}}{p_{in}}\right)}^{\frac{\gamma +1}{\gamma }}\right)\right)}^{\frac{1}{\gamma +1}}$
*p*	$e_{0}\rho_{in}^{\gamma }$	$e_{0}\rho_{out}^{\gamma }$	$e_{0}\rho_{in}^{\gamma }{\left(1-\frac{x}{w}\left(1-{\left(\frac{\rho_{out}}{\rho_{in}}\right)}^{\gamma +1}\right)\right)}^{\frac{\gamma }{\gamma +1}}$
ρ	$\rho_{in}$	$\rho_{out}$	$\rho_{in}{\left(1-\frac{x}{w}\left(1-{\left(\frac{\rho_{out}}{\rho_{in}}\right)}^{\gamma +1}\right)\right)}^{\frac{1}{\gamma +1}}$

**Table 4:** Overview of stationary solutions to Neumann BVPs.

*u*	$u_{\mathrm{in}}^{\prime }$	$u_{\mathrm{out}}^{\prime }$	u(x)
*p*	$p_{in}^{\prime }$	−∞	$\frac{\gamma +1}{\gamma }w\left(-p_{in}^{\prime }\right){\left(1-\frac{x}{w}\right)}^{\frac{\gamma }{\gamma +1}}$
ρ	$\rho^{\prime }\left(0\right)$	−∞	${\left(\frac{\gamma +1}{\gamma }w\left(-\frac{p_{in}^{\prime }}{e_{0}}\right)\right)}^{\frac{1}{\gamma }}{\left(1-\frac{x}{w}\right)}^{\frac{1}{\gamma +1}}$
*p*	$p^{\prime }\left(0\right)$	−∞	$e_{0}{\left(\left(\gamma +1\right)w\left(-\rho_{in}^{\prime }\right)\right)}^{\gamma }{\left(1-\frac{x}{w}\right)}^{\frac{\gamma }{\gamma +1}}$
ρ	$\rho_{in}^{\prime }$	−∞	$\left(\gamma +1\right)w\left(-\rho_{in}^{\prime }\right){\left(1-\frac{x}{w}\right)}^{\frac{1}{\gamma +1}}$
*p*	$p_{in}^{\prime }$	$p_{out}^{\prime }$	$\frac{\frac{\gamma +1}{\gamma }w\left(-p_{in}^{\prime }\right)}{1-{\left(\frac{p_{in}^{\prime }}{p_{out}^{\prime }}\right)}^{\gamma +1}}{\left(1-\frac{x}{w}\left(1-{\left(\frac{p_{in}^{\prime }}{p_{out}^{\prime }}\right)}^{\gamma +1}\right)\right)}^{\frac{\gamma }{\gamma +1}}$
ρ	$\rho^{\prime }\left(0\right)$	$\rho^{\prime }\left(w\right)$	${\left(\frac{\frac{\gamma +1}{\gamma }w\left(-\frac{p_{in}^{\prime }}{e_{0}}\right)}{1-{\left(\frac{p_{in}^{\prime }}{p_{out}^{\prime }}\right)}^{\gamma +1}}\right)}^{\frac{1}{\gamma }}{\left(1-\frac{x}{w}\left(1-{\left(\frac{p_{in}^{\prime }}{p_{out}^{\prime }}\right)}^{\gamma +1}\right)\right)}^{\frac{1}{\gamma +1}}$
*p*	$p^{\prime }\left(0\right)$	$p^{\prime }\left(w\right)$	$e_{0}{\left(\frac{\left(\gamma +1\right)w\left(-\rho_{in}^{\prime }\right)}{1-{\left(\frac{\rho_{in}^{\prime }}{\rho_{out}^{\prime }}\right)}^{\frac{\gamma +1}{\gamma }}}\right)}^{\gamma }{\left(1-\frac{x}{w}\left(1-{\left(\frac{\rho_{in}^{\prime }}{\rho_{out}^{\prime }}\right)}^{\frac{\gamma +1}{\gamma }}\right)\right)}^{\frac{\gamma }{\gamma +1}}$
ρ	$\rho_{in}^{\prime }$	$\rho_{out}^{\prime }$	$\frac{\left(\gamma +1\right)w\left(-\rho_{in}^{\prime }\right)}{1-{\left(\frac{\rho_{in}^{\prime }}{\rho_{out}^{\prime }}\right)}^{\frac{\gamma +1}{\gamma }}}{\left(1-\frac{x}{w}\left(1-{\left(\frac{\rho_{in}^{\prime }}{\rho_{out}^{\prime }}\right)}^{\frac{\gamma +1}{\gamma }}\right)\right)}^{\frac{1}{\gamma +1}}$

**Table 5:** Overview of nonlinear regression parameters, isobaric heat capacities at 25 °C [43], and calculated heat capacities based on the polytropic exponent.

Gas	$b\;\left[\mathrm{Barrer}\right]$	$\gamma \;\left[-\right]$	$c_{p}\;\left[\frac{J}{\mathrm{mol}\cdot K}\right]$	$c\;\left[\frac{J}{\mathrm{mol}\cdot K}\right]$
CH${}_{4}$	2.249	2.661	35.56	30.553
CO${}_{2}$	1.371	3.489	36.45	30.465
N${}_{2}$	1.556	3.028	29.18	25.080
H${}_{2}$	4.772	6.345	28.88	27.325
H${}_{2}^{*}$	4.534	4.370	28.88	26.413
C${}_{3}$H${}_{8}$	1.551	3.219	73.85	70.104

## Data Availability

Processed data is contained within the article, raw measured data is available on demand from L.M.

## Nomenclature

Symbol	Unit	Description
$A_{M}$	$m^{2}$	Membrane area
*b*	Barrer	Power-law set-point
*B*	Barrer	Membrane gas permeability
*C*	$J\cdot K^{-1}$	Heat capacity
$C_{P}$	$J\cdot K^{-1}$	Heat capacity, isobaric
$C_{V}$	$J\cdot K^{-1}$	Heat capacity, isochoric
*D*	$m^{2}\cdot s^{-1}$	Diffusion coefficient
$e_{0}$	$m^{2}\cdot s^{-2}$	Specific energy
*k*	$m^{2}$	Darcy’s permeability
*m*	kg	Mass
*M*	$\mathrm{kg}\cdot {\mathrm{mol}}^{-1}$	Molar mass
*n*	mol	Molar amount
*p*	Pa	Pressure
$\vec{q}$	$m^{3}\cdot s^{-1}$	Volume flux
*Q*	J	Heat
*R*	$J\cdot K^{-1}\cdot {\mathrm{mol}}^{-1}$	Universal gas constant
*T*	K	Temperature
*U*	J	Internal energy
$\vec{v}$	$m\cdot s^{-1}$	Velocity vector field
*W*	J	Work
α	–	Selectivity
γ	–	Polytropic exponent/index
ε	–	Porosity
μ	Pa·s	Dynamic viscosity
ρ	$\mathrm{kg}\cdot m^{-3}$	Density

## Appendix A. Derivation of Porous Medium Equation

Porous Medium Equation as described, e.g., by Vazquez [41] is any equation of the form

(A1) $$\frac{\partial u}{\partial t}=\Delta \left(u^{\Gamma }\right),\;\Gamma >1$$

posed for a scalar quantity u(x,t), typically in one or three spatial dimensions. He sets the course of derivation for our application; however, he further studies only the general case, as from mathematician’s point of view the multiplicative constants can be easily scaled out. We are more interested in specific forms of PME for pressure and density and the relation between them, thus we derive both of them here, providing useful insight.

### Appendix A.1. Prerequisites

To set some ground for the derivation we state the continuity equation and Darcy’s law of diffusion. The third equation, which due to lack of nomenclature consensus we will call a polytropic state equation, we derive properly as it is the source of nonlinearity in PME and provides a possible physical interpretation. These three together form the foundation of the derivation of PME.

Continuity equation in porous media, especially the introduction and formalization of medium porosity as the mean ratio of void and solid regions in a control volume, has been thoroughly established by S. Whitaker [44]. We will use the formulation

(A2) $$\varepsilon \frac{\partial \rho }{\partial t}+\nabla \cdot \left(\rho \vec{v}\right)=0,$$

where ρ is the density of gas, $\vec{v}$ is the velocity vector field, ε is the porosity of membrane, and ∇· is the divergence operator.

Darcy’s law of diffusion describes the flow of fluid through porous medium. It has been theoretically derived from Stokes equation [45], for consistency we once again refer to the work of Whitaker [46]. We will use the formulation

(A3) $$\mu \vec{v}=-k\nabla p,$$

where μ stands for the dynamic viscosity of gas, *k* is Darcy’s permeability, and *p* is the gas pressure.

Polytropic state equation follows from the first law of thermodynamics and state equation of ideal gas, accompanied by Mayer’s relation and exploitation of some properties of ideal gas.

We begin with the first law of thermodynamics in differential form,

(A4) δQ=dU+δW,

where δQ is the heat obtained by the system, dU is the inner energy differential, and δW is the work done by the system on its surroundings (we follow Clausius sign convention).

We introduce a change of temperature dT in our system. The change of inner energy of ideal gas is

(A5) $$\;dU=C_{V}dT,$$

where $C_{V}$ is the heat capacity at constant volume. The total heat given to the system introduces some unknown heat capacity *C*,

(A6) δQ=CdT.

For the work term only pressure-volume work is considered,

(A7) δW=pdV.

Substituting back to (A4) we can express the temperature differential,

(A8) $$CdT=C_{V}dT+pdV\Rightarrow dT=\frac{pdV}{C-C_{V}}.$$

Let us do the same for the state equation of ideal gas. For *R* being the universal gas constant, in differential form we have

(A9) $$Vdp+pdV=nRdT\Rightarrow dT=\frac{Vdp+pdV}{nR}.$$

Combining (A8) and (A9) in a single equation yields

(A10) $$\frac{pdV}{C-C_{V}}=\frac{Vdp+pdV}{nR}.$$

Firstly we scale out *n* by introducing molar heat capacities $c=\frac{C}{n}$ and $c_{V}=\frac{C_{V}}{n}$,

(A11) $$\frac{pdV}{n\left(c-c_{V}\right)}=\frac{Vdp+pdV}{nR}.$$

Now recall Mayer’s relation: $c_{p}=c_{V}+R$ for $c_{p}$ being the molar heat capacity of isobaric process. We can now rewrite the differential form as a separable differential equation,

(A12) $$\begin{array}{cc} RpdV & =\left(c-c_{V}\right)\left(Vdp+pdV\right)\\ \left(c-c_{V}\right)Vdp & =-\left(c-c_{V}-R\right)pdV \end{array}$$

which can be solved for *p*,

(A13) $$p=KV^{-\frac{c-c_{p}}{c-c_{V}}},\;K>0.$$

At last we introduce density via $V=\frac{m}{\rho }$,

(A14) $$p=K{\left(\frac{m}{\rho }\right)}^{-\frac{c-c_{p}}{c-c_{V}}}=Km^{-\frac{c-c_{p}}{c-c_{V}}}\rho^{\frac{c-c_{p}}{c-c_{V}}},\;K>0$$

where *m*, as a conserved quantity, is constant. The term $Km^{-\frac{c-c_{p}}{c-c_{V}}}$ has a unit of $\left[m^{2}\cdot s^{-2}\right]$ and can be thought of as specific energy (energy per unit mass). Let us hide the term inside new constant, $e_{0}>0$. The exponent is called the polytropic exponent or polytropic index, we denote it $\gamma =\frac{c-c_{p}}{c-c_{V}}$. Using these two substitutions, we get the final form of the polytropic state equation,

(A15) $$p=e_{0}\rho^{\gamma }.$$

Porous medium equation (PME) is derived from these three laws: continuity Equation (A2), Darcy’s Law (A3), and polytropic state Equation (A15). We will derive PME in terms of pressure and density in three different forms:

- Laplacian form, where the Laplace operator acts on nonlinear expression of our quantity—in this form we can easily compare PME to linear heat equation;
- Divergence form, which is numerically treatable with Finite Volume Method;
- Linearized form, where both differential operators (Laplacian and gradient) act on linear expression of our quantity—in this form the equation can be treated with Finite Differences Method.

### Appendix A.2. Porous Medium Equation for Pressure

We start by expressing the time derivative from (A2),

(A16) $$\frac{\partial \rho }{\partial t}=-\frac{1}{\varepsilon }\nabla \cdot \left(\rho \vec{v}\right).$$

the velocity from (A3),

(A17) $$\vec{v}=-\frac{k}{\mu }\nabla p,$$

and the density from (A15),

(A18) $$\rho ={\left(\frac{p}{e_{0}}\right)}^{\frac{1}{\gamma }}.$$

Plugging (A17) and (A18) into (A16), we obtain a nonlinear PDE,

(A19) $$\frac{\partial }{\partial t}\left({\left(\frac{p}{e_{0}}\right)}^{\frac{1}{\gamma }}\right)=-\frac{1}{\varepsilon }\nabla \cdot \left({\left(\frac{p}{e_{0}}\right)}^{\frac{1}{\gamma }}\left(-\frac{k}{\mu }\nabla p\right)\right).$$

Focusing on the left-hand side, we factor out the constant and apply chain rule,

(A20) $$\frac{\partial }{\partial t}\left({\left(\frac{p}{e_{0}}\right)}^{\frac{1}{\gamma }}\right)={e_{0}}^{-\frac{1}{\gamma }}\;\frac{\partial }{\partial t}\left(p^{\frac{1}{\gamma }}\right)={e_{0}}^{-\frac{1}{\gamma }}\;\frac{1}{\gamma }\;p^{\frac{1}{\gamma }-1}\;\frac{\partial p}{\partial t}.$$

On the right-hand side, we factor out all the constants as well,

(A21) $$-\frac{1}{\varepsilon }\nabla \cdot \left({\left(\frac{p}{e_{0}}\right)}^{\frac{1}{\gamma }}\left(-\frac{k}{\mu }\nabla p\right)\right)=\frac{k}{\varepsilon \mu }\;{e_{0}}^{-\frac{1}{\gamma }}\;\nabla \cdot \left(p^{\frac{1}{\gamma }}\nabla p\right).$$

Now we convert the exponents to a common denominator,

(A22) $${e_{0}}^{-\frac{1}{\gamma }}\;\frac{1}{\gamma }\;p^{\frac{1-\gamma }{\gamma }}\;\frac{\partial p}{\partial t}=\frac{k}{\varepsilon \mu }\;{e_{0}}^{-\frac{1}{\gamma }}\;\nabla \cdot \left(p^{\frac{1}{\gamma }}\nabla p\right),$$

multiply by appropriate factor to separate the time derivative,

(A23) $$\frac{\partial p}{\partial t}=\frac{k}{\varepsilon \mu }\;\gamma \;p^{\frac{\gamma -1}{\gamma }}\nabla \cdot \left(p^{\frac{1}{\gamma }}\nabla p\right),$$

and we are looking at the divergence form of the pressure PME.

As a side step, applying chain rule on a purposefully chosen expression yields

(A24) $$\begin{array}{cc} \Delta \left(p^{1+\frac{1}{\gamma }}\right) & =\nabla \cdot \nabla \left(p^{1+\frac{1}{\gamma }}\right)=\nabla \cdot \left(\nabla \left(p^{\frac{\gamma +1}{\gamma }}\right)\right)\\ \; & =\nabla \cdot \left(\left(1+\frac{1}{\gamma }\right)p^{\frac{1}{\gamma }}\nabla p\right)=\frac{\gamma +1}{\gamma }\nabla \cdot \left(p^{\frac{1}{\gamma }}\nabla p\right). \end{array}$$

We can now express the divergence part of (A23) from (A24),

(A25) $$\frac{\gamma }{\gamma +1}\Delta \left(p^{\frac{\gamma +1}{\gamma }}\right)=\nabla \cdot \left(p^{\frac{1}{\gamma }}\nabla p\right),$$

which, plugged in (A23), gives us the Laplacian form,

(A26) $$\frac{\partial p}{\partial t}=\frac{k}{\varepsilon \mu }\;\frac{\gamma^{2}}{\gamma +1}\;p^{\frac{\gamma -1}{\gamma }}\;\Delta \left(p^{\frac{\gamma +1}{\gamma }}\right).$$

Let us resort back to (A23) and actually perform the divergence operator. By following the product rule, we get

(A27) $$\nabla \cdot \left(p^{\frac{1}{\gamma }}\nabla p\right)=\frac{1}{\gamma }\;p^{\frac{1}{\gamma }-1}\nabla p\cdot \nabla p+p^{\frac{1}{\gamma }}\nabla \cdot \nabla p=p^{\frac{1-\gamma }{\gamma }}\left(\frac{1}{\gamma }{∥\nabla p∥}^{2}+p\Delta p\right),$$

which, plugged into (A23) results in cancellation of multiplicative pressure term,

(A28) $$\begin{array}{cc} \frac{\partial p}{\partial t} & =\frac{k}{\varepsilon \mu }\;\gamma \;p^{\frac{\gamma -1}{\gamma }}\nabla \cdot \left(p^{\frac{1}{\gamma }}\nabla p\right)=\frac{k}{\varepsilon \mu }\;\gamma \;p^{\frac{\gamma -1}{\gamma }}p^{\frac{1-\gamma }{\gamma }}\left(\frac{1}{\gamma }{∥\nabla p∥}^{2}+\Delta p\right)\\ \; & =\frac{k}{\varepsilon \mu }\;\left({∥\nabla p∥}^{2}+\gamma p\Delta p\right), \end{array}$$

resulting in the linearized form.

### Appendix A.3. Porous Medium Equation for Density

For density, the derivation follows the same steps as for pressure. We start by expressing the time derivative from (A2),

(A29) $$\frac{\partial \rho }{\partial t}=-\frac{1}{\varepsilon }\nabla \cdot \left(\rho \vec{v}\right).$$

the velocity from (A3),

(A30) $$\vec{v}=-\frac{k}{\mu }\nabla p,$$

and the polytropic state Equation (A15) remains as it is,

(A31) $$p=e_{0}\rho^{\gamma }.$$

Plugging (A31) into (A30), we get

(A32) $$\vec{v}=-\frac{k}{\mu }\nabla \left(e_{0}\rho^{\gamma }\right)=-\frac{ke_{0}}{\mu }\nabla \left(\rho^{\gamma }\right),$$

and subsequently plugging (A32) into (A29) we get

(A33) $$\frac{\partial \rho }{\partial t}=-\frac{1}{\varepsilon }\nabla \cdot \left(\rho \left(-\frac{ke_{0}}{\mu }\right)\nabla \left(\rho^{\gamma }\right)\right)=\frac{ke_{0}}{\varepsilon \mu }\nabla \cdot \left(\rho \nabla \left(\rho^{\gamma }\right)\right).$$

Following the chain rule,

(A34) $$\frac{\partial \rho }{\partial t}=\frac{ke_{0}}{\varepsilon \mu }\nabla \cdot \left(\rho \nabla \left(\rho^{\gamma }\right)\right)=\frac{ke_{0}}{\varepsilon \mu }\nabla \cdot \left(\rho \gamma \rho^{\gamma -1}\nabla \rho \right)=\frac{ke_{0}}{\varepsilon \mu }\gamma \nabla \cdot \left(\rho^{\gamma }\nabla \rho \right),$$

we obtain the divergence form.

Side-stepping to a purposefully chosen expression, we perform the divergence,

(A35) $$\Delta \left(\rho^{\gamma +1}\right)=\nabla \cdot \nabla \left(\rho^{\gamma +1}\right)=\nabla \cdot \left(\left(\gamma +1\right)\rho^{\gamma }\nabla \rho \right)=\left(\gamma +1\right)\nabla \cdot \left(\rho^{\gamma }\nabla \rho \right).$$

We express the divergence part of (A34) from (A35),

(A36) $$\frac{1}{\gamma +1}\Delta \left(\rho^{\gamma +1}\right)=\nabla \cdot \left(\rho^{\gamma }\nabla \rho \right),$$

which, plugged in (A34), yields the Laplacian form,

(A37) $$\frac{\partial \rho }{\partial t}=\frac{ke_{0}}{\varepsilon \mu }\frac{\gamma }{\gamma +1}\Delta \left(\rho^{\gamma +1}\right).$$

Returning back to (A34), we can perform the divergence operator on the product,

(A38) $$\begin{array}{cc} \frac{\partial \rho }{\partial t} & =\frac{ke_{0}}{\varepsilon \mu }\gamma \nabla \cdot \left(\rho^{\gamma }\nabla \rho \right)=\frac{ke_{0}}{\varepsilon \mu }\gamma \left(\gamma \rho^{\gamma -1}{∥\nabla \rho ∥}^{2}+\rho^{\gamma }\Delta \rho \right)\\ & =\frac{ke_{0}}{\varepsilon \mu }\rho^{\gamma -1}\left(\gamma^{2}{∥\nabla \rho ∥}^{2}+\gamma \rho \Delta \rho \right), \end{array}$$

resulting in linearized form of PME.
